# The Sufferings of Christ

**Published:** 1888-05-05

**Authors:** 


### WORDS OF CONSOLATION.

[Communications for this column should be addressed "Chaplain," care of the Editor of THE HOSPITAL, who will gratefully welcome any suggestions or inquiries from matrons, nurses, and the staff generally.]

### LXXV.—THE SUFFERINGS OF CHRIST.

*For Reading to the Sick.*

SEEING that "the blood is the life," and knowing as we do that participation in the life of Christ is essential to salvation, it may be as well to refer to a few well-known passages of Scripture which must be taken in this light if they are to be fully understood. "The blood of Jesus Christ cleanseth us from all sin" (1 John i. 7). What is meant here? Is it the physical and material drops of blood which fell from the cross? Nay, much rather the blood which is the life, given unto us by the living Saviour. The cleansing needed is in the present; it must be continual; so also must be the precious blood which effects the cleansing. We need cleansing in addition to forgiveness. "He is faithful and just to forgive us our sins, and to cleanse us from all unrighteousness" (1 John i. 9). Again, "How much more shall the blood of Christ, who through the Eternal Spirit offered himself without spot to God, purge your conscience from dead works to serve the living God?" (Heb. ix. 14). What is meant? The drops drunk in once by the earth on Calvary! That shedding of blood made a propitiation for sin: but it cannot assist in purging out a present disease. The purging so necessary now can only be effected by a present remedy.

The Bible contains two ideas under the name of blood: (1) As shed in sacrifice; (2) as offered with a view to its being communicated as a principle of life. In this latter case the power is of course extended and becomes active beyond death. Only through union with a living Christ can we now wash our robes and make them white in the blood of the Lamb. Now we commend to your attention the words of a well-known hymn, which fully expresses the truth concerning the precious blood of Christ, provided you remember that "the blood is the life," a very present antidote to the poison of sin.

GLORY be to Jesus,
Who, in bitter pains,
Poured for me the life-blood
From His sacred veins!

Grace and life eternal
In that blood I find,
Blest be his compassion
Infinitely kind!

Blest through endless ages
Be the precious stream,
Which from endless torments
Did the world redeem!

Abel's blood for vengeance
Pleaded to the skies;
But the blood of Jesus
For our pardon cries.

Oft as it is sprinkled
On our guilty hearts,
Satan in confusion
Terror-struck departs;

Oft as earth exulting
Wafts its praise on high,
Angel-hosts rejoicing
Make their glad reply.

Lift ye then your voices;
Swell the mighty flood;
Louder still and louder
Praise the precious Blood. Amen.

